# 2432 The relationship between cognitive functioning and abnormal eating behavior in behavioral variant frontotemporal dementia

**DOI:** 10.1017/cts.2018.189

**Published:** 2018-11-21

**Authors:** Vidyulata Kamath, Grace-Anna Chaney, Chiadi Onyike

**Affiliations:** Johns Hopkins University School of Medicine

## Abstract

OBJECTIVES/SPECIFIC AIMS: Abnormal eating behavior is a core and distinguishing diagnostic feature of behavioral variant frontotemporal dementia (bvFTD) that differentiates it from other neurodegenerative disorders and late-life psychiatric conditions. Though it has been proposed that hyperphagia in bvFTD results from cognitive dysfunction, the observation of altered sweet preferences and food foraging indicate that bvFTD is accompanied by fundamental dietary changes associated with hypothalamic and insular atrophy. In the current study, we examined how cognitive dysfunction contributes to abnormal feeding behavior in bvFTD. METHODS/STUDY POPULATION: We analyzed first-visit eating and neuropsychological data from the National Alzheimer’s Coordinating Center database (7 centers; September 2017 data freeze) in a subset of bvFTD patients with clinician-rated characterization of disturbed feeding severity. Group differences in cognitive domains of attention, processing speed, language, memory, and executive functioning were examined between patients with abnormal eating behavior (n=59) and a demographically-matched sample of patients with normal feeding behavior (n=60). Group differences in informant-reported empathy, behavioral inhibition, and depressive symptoms were also examined. RESULTS/ANTICIPATED RESULTS: Cognitive profiles in bvFTD patients did not vary as a function of disturbed feeding behavior. In a subset of cases pathologically-confirmed at autopsy, processing speed was better in cases with abnormal feeding behavior. No significant group differences were found for behavioral indices. DISCUSSION/SIGNIFICANCE OF IMPACT: These findings suggest that cognitive dysfunction is not the sole driver of abnormal eating behavior in bvFTD. Future studies with comprehensive characterization of feeding behavior, cognition and physiological/neuroimaging indices are warranted.

